# Supplementary material for: Matching sensor ontologies through siamese neural networks without using reference alignment
Source: PeerJ Comput Sci. 2021 Jun 18;7:e602. doi: 10.7717/peerj-cs.602 (PMC8237319; doi:10.7717/peerj-cs.602)
Supplement: Supplemental Information 1 [file peerj-cs-07-602-s001.zip › 265/onto.html]

Author: Nick Knouf <nknouf@mit.edu>  
Contributor: Antoine Zimmermann <antoine.zimmermann@inrialpes.fr>, Jérôme Euzenat,   
Date: 08/06/2005  
Version: $Id: onto-flat.rdf,v 1.15 2011/05/31 15:37:47 euzenat Exp $

## Classes

**http://www.w3.org/1999/02/22-rdf-syntax-ns#List** (, *)*


**http://xmlns.com/foaf/0.1/Person** (, *)*


**http://xmlns.com/foaf/0.1/Organization** (, *)*


**sqdsq** (, *)*
:   **hazdn** (, *)*


    **dzajndsq** (, *)*


    **sqxsqkd** (, *)*


    **zdzaudbd** (, *)*


    **dzqndbzq** (, *)*


    **vccfsq** (, *)*


    **dcsqdcsqd** (, *)*


    **deqdxcsqcsq** (, *)*


    **scds** (, *)*


    **dcsqdsq** (, *)*


    **xsqlknk** (, *)*


    **zqdzqzai** (, *)*


    **wxsdxqsdh** (, *)*


    **zdqssqdb** (, *)*


    **dqzdxdcsqj** (, *)*
    :   **sdcsqhyz** (, *)*


        **azzauhuie** (, *)*

    **zeadza** (, *)*


    **dscdscg** (, *)*

**qsdsquj** (, *)*


**zqedzbx** (, *)*


**qzddj** (, *)*


**izxnquo** (, *)*
:   super: *http://xmlns.com/foaf/0.1/Organization*  


    **zauio** (, *)*


    **zadazxn** (, *)*

**dsqdbz** (, *)*
:   super: *http://www.w3.org/1999/02/22-rdf-syntax-ns#List*

**dnqsb** (, *)*


**dzshyuua** (, *)*

## Properties

**http://www.w3.org/1999/02/22-rdf-syntax-ns#first**: http://www.w3.org/1999/02/22-rdf-syntax-ns#List -> \_ *()*


**http://www.w3.org/1999/02/22-rdf-syntax-ns#rest**: http://www.w3.org/1999/02/22-rdf-syntax-ns#List -> http://www.w3.org/1999/02/22-rdf-syntax-ns#List *()*

**http://purl.org/dc/elements/1.1/creator**\_ -> \_ *()*


**http://purl.org/dc/elements/1.1/contributor**\_ -> \_ *()*


**http://purl.org/dc/elements/1.1/description**\_ -> \_ *()*


**http://purl.org/dc/elements/1.1/date**\_ -> \_ *()*


**http://xmlns.com/foaf/0.1/firstName**\_ -> \_ *()*


**#lastName**\_ -> \_ *()*


**http://xmlns.com/foaf/0.1/name**\_ -> \_ *()*

## Individuals

<rdf:List@ttp://www.w3.org/1999/02/22-rdf-syntax-ns#nil>

---

Generated by OWL2HTML
